# Ankylosis of the Jaw

**Published:** 1919

**Authors:** 


***Ankylosis of the Jaw.*** By Dr. M. S. Henderson and Dr. G. B. New. *Surgery, Gynecology and Obstetrics*, November, 1918.

It is not believed by the authors that the good results obtainable by arthroplasty of the temporo-maxillary joint are generally understood, and they review twenty-three cases of ankylosis of the lower jaw treated at the Mayo Clinic.

According to location of the fixation they are :

(1) the articular type, involving only the joint; (2) the extra-articular, such as scarring in the muscles of the cheek or temporal region; (3) articular-extra-articular, where the cause lies both within and without the joint. Fifteen belonged to the first class, five to second, and three to the third class of cases.

Articular ankylosis is due either to traumatism or infection extending into the joint directly or by the blood-stream. Blair states that fifty per cent. of cases are due to trauma on the chin, and Murphy believes they are due to extension from middle ear infection into the glenoid cavity, or over the base of the zygoma to the joint. The authors' percentage of trauma was 20 and only one case resulted from middle ear infection.

The condition usually occurs early in life, hence a clear history is difficult to get. Nearly fifty per cent. of all ankyloses of the jaw occur in the first decade. In the articular-extra-articular variety more than forty five per cent. occur between 21 and 30.

Any fixation of the epiphysis of the condyle interferes with the development of the jaw on the side affected. The center of the condyle does not unite with the ramus until the age of fifteen, and injury will cause lack of development of that side. Fibrous adhesions cause ankylosis in that type, while the condyle and zygoma are sometimes obliterated by bony adhesions. In the extra-articular type the scarring of muscle, temporal, masseter, etc., limits the movement of the jaw.

A photograph is shown illustrating "unilateral ankylosis of the lower jaw" before full development, and the result of operation. The ramus on the affected side is shortened, and when the mouth is opened it turns toward that side. X-ray is useful in differentiating the type present. In bony ankylosis a definite diagnosis can only be made where the deformity occurred early in life. Nine of the cases cited were bony, and six fibrous. In two patients having no deformity diagnosis was based on the divergence of the jaw when opened.

Excision has been the surgical procedure for years and is still the basic principle of operation. Dr. Murphy of Chicago employed a flap of autogenous fat or fascia in the joint with good results, and Dr. Baer advises chromicized pig's bladder for the same purpose.

The facial nerve and internal maxillary and superficial temporal arteries are the structures which must be familiar and constantly borne in mind. Some little bleeding may occur, but packing with a hot salt sponge controls this when necessary.

The triangular area with the base upward, which lies over the temporo-maxillary joint, is practically without important anatomical structures. Approach should be from above, and the lower portion of the zygoma must be sacrificed, although a bridge is left to obviate deformity. Incision is curved, some two inches in length, and the skin flap is dissected free. An incision is made directly over the zygoma and the fascia retracted downward to expose the zygoma and the joint. The flap is turned back and held by a self-retaining mastoid retractor. It is well for a skilled surgeon to do the retraction. The part of the zygoma directly over the joint area is now removed, with care to avoid injury of the external auditory canal. A small bridge of the zygoma must be left in place. The condyle is now exposed and removed with a chisel gouge, being carefully chipped off in small pieces to avoid injury to the internal maxillary artery. In some cases the bony ankylosis involves the ramus and the coronoid process. It is then necessary to remove a large quantity of bone, and the space left must be at least half an inch in width. Where the coronoid process is involved, sufficient must be removed, by working forward, to permit free motion. No foreign material of any kind is placed between the mandible and temporal bone. When the bone is removed and motion secured, the wound is closed.

If sufficient motion has not resulted and there is no facial deformity, it is well to examine the opposite side for ankylosis. The trouble, however, may lie in the muscles. Gently forcing the mouth open each day with a mouth-spreader will help this condition. The patient tries out his muscles also by chewing gum and tough meat. The operations cited have resulted in separation of the jaws from one to one and three-quarters of an inch.

The extra-articular cases have given best results by forcible stretching under ether.

The essential points brought out are in part as follows :

1. Removing sufficient bone to make a half-inch space between skull and ramus, obtaining a stable functionating joint.

2. An incision that gives good exposure of the joint and does not injure the facial nerve.

3. Approaching the joint from above by removing part of the zygoma.

